# Impact of sit-stand desks at work on energy expenditure, sitting time and cardio-metabolic risk factors: Multiphase feasibility study with randomised controlled component

**DOI:** 10.1016/j.pmedr.2018.11.012

**Published:** 2018-11-26

**Authors:** Eleni Mantzari, Catherine Galloway, Katrien Wijndaele, Soren Brage, Simon J. Griffin, Theresa M. Marteau

**Affiliations:** a Behaviour and Health Research Unit, University of Cambridge, Cambridge, UK; b MRC Epidemiology Unit, University of Cambridge, Cambridge, UK

**Keywords:** Sit-stand desks, Standing desks, Height-adjustable desks, Sitting, Standing, Energy expenditure, Sedentary behaviour, Feasibility study

## Abstract

Uncertainties remain about the overall effect of sit-stand desks for reducing prolonged sitting among office-based workers. This study assessed the feasibility of a randomised controlled trial of the impact of workplace sit-stand desks on overall energy expenditure, sitting time and cardio-metabolic outcomes. It involved four phases: Phase I: online survey; Phase II: workspace auditing; Phase III: randomised intervention (provision of sit-stand desks at work for 3 months); Phase IV: qualitative component. Participants were offıce-based employees of two companies in Cambridge, England. Among Phase I participants interested in the trial, 100 were randomised to Phase II. Of those with workspaces suitable for sit-stand desks, 20 were randomised to Phase III. Those allocated to the intervention completed Phase IV. Outcomes included: trial participation interest, desk-type (full desks/desk mounts) and assessment location (work/laboratory/home) preferences (Phase I); proportion of workspaces permitting sit-stand desk installation (Phase II); energy expenditure, sitting time and cardio-metabolic outcomes (Phase III); study participation experiences (Phase IV). Data were collected between May 2015 and December 2016. Recruitment and trial implementation were feasible: 92% of survey respondents expressed participation interest; 80% of workspaces could accommodate sit-stand desks; assessments were done in workplaces, preferred by 71%. Sit-stand desk provision reduced workplace sitting time by 94 min/day (95% CI 17.7–170.7). Their impact on energy expenditure and cardio-metabolic outcomes is unclear. The results confirm the feasibility of a trial assessing sit-stand desks' impact on energy expenditure, sitting time and cardio-metabolic outcomes, which should reduce uncertainty concerning the intervention's potential to reduce the health risks of prolonged sitting.

**Trial registration** ISRCTN44827407.

## Highlights

• The overall effect of sit-stand desks for reducing sitting at work is uncertain.
• A trial of sit-stand desks on energy expenditure, sitting and health factors is feasible.
• Preliminary evidence suggests sit-stand desks' potential to reduce sitting at work.
• Research should assess sit-stand desks' likely compensation effect and health impact.

## 1 Introduction

There is strong evidence that physical inactivity increases the risk of many health conditions, including coronary heart disease and type 2 diabetes (Lee et al., 2012). Recent findings suggest that high levels of sedentary behaviour i.e. any waking behaviour characterised by low energy expenditure while sitting or reclining (Tremblay et al., 2017), may be an independent risk factor for ill health (Chau et al., 2013; Wilmot et al., 2012; Ekelund et al., 2016; Brocklebank et al., 2015; Biswas et al., 2015). For example, compared with those who sit the least, those who sit the most have over twice the risk of developing type 2 diabetes and cardiovascular disease (Wilmot et al., 2012) and a 13% increased risk of cancer incidence (Biswas et al., 2015). Furthermore, each additional hour of daily sitting has been associated with an 2% increased risk of all-cause mortality, a rate which more than doubles for adults sitting more than 7 h a day (Chau et al., 2013). A recent meta-analysis including data from more than one million adults indicated that the risks associated with sitting are only mitigated by more than an hour per day of at least moderate-intensity physical activity (Ekelund et al., 2016), double the amount recommended in current activity guidelines. Uninterrupted sitting may be particularly problematic, being linked with unfavourable cardio-metabolic profiles, regardless of total sitting time (Peddie et al., 2013; Chastin et al., 2015).

Adults in middle- and high-income countries increasingly spend the majority of their days in sedentary behaviour, mostly at work, often in uninterrupted bouts (Peddie et al., 2013; Ng & Popkin, 2012; Parry & Straker, 2013). High levels of sedentary behaviour at work are rarely compensated for during leisure time i.e. through increased physical activity levels and/or reduced sitting time (Parry & Straker, 2013; Clemes et al., 2014). Given that office workers are one of the largest occupational groups in high- and middle-income countries (Offıce for National Statistics, 2011), decreasing their sedentary behaviour could have important public health benefits (Healy et al., 2012). One possible intervention is to provide adjustable sit-stand desks, allowing work postures to vary between sitting and standing. Such changes to the work environment have received considerable interest but the quality of the evidence for their impact is limited due to suboptimal study designs, including a lack of randomisation (Alkhajah et al., 2012; Adeleke et al., 2017; Chau et al., 2016; Mansoubi et al., 2015; Healy et al., 2013; Straker et al., 2013). Existing randomised trials exhibit a number of limitations including: i. lack of control group (Buman et al., 2017); ii. Possible residual confounding (Buman et al., 2017; Dutta et al., 2014; Sandy, 2016); iii. Use of multicomponent interventions (Ellegast et al., 2012; Healy et al., 2016; O'Connell et al., 2015; Danquah et al., 2017; Hedge, 2004) impeding isolation of sit-stand desk effects; iv. Use of cluster designs resulting in recruitment bias and reduced power (Buman et al., 2017; Healy et al., 2016), and v. small samples (Tobin et al., 2016; Schwartz et al., 2016; Hall et al., 2015; Graves et al., 2015; Neuhaus et al., 2014; DM et al., 2014; Radas et al., 2013). Furthermore, many existing trials include measures of sitting time that are i. subjective (Sandy, 2016; Hedge, 2004; Graves et al., 2015), ii. Inadequately validated (Dutta et al., 2014; Schwartz et al., 2016); and/or iii. Unable to discriminate between sitting and standing (Dutta et al., 2014; Schwartz et al., 2016; Radas et al., 2013). In addition, there is a lack of robust estimation of the potential compensation effects of sit-stand desks—i.e. their potential to increase sitting time and/or energy intake from food and drink and alter physical activity patterns outside work— as well as of their cardio-metabolic health impacts (Buman et al., 2017; Dutta et al., 2014; Healy et al., 2016; Schwartz et al., 2016; DM et al., 2014).

There are substantial evidence gaps concerning the potential health benefits (MacEwen et al., 2015) and/or harms of providing office-based workers with sit-stand desks. This uncertainty is exacerbated by lack of assessments of key outcomes relevant to their use. One such outcome is overall energy expenditure. Precise quantification of the impact of sit-stand desks on energy expenditure, both in and outside the workplace, is essential to fully gauge their potential cardio-metabolic health impacts, including their potential for harm. The evidence for claims that sit-stand desks increase energy expenditure is equivocal (MacEwen et al., 2015). Another outcome not examined by most trials, is longer-term behaviour change (i.e. ≥6 months after desk installation), which is essential for estimating the sustainability of any observed effects. The evidence from the few trials with long-term assessments (i.e. ≥6 months after desk installation) (Buman et al., 2017; O'Connell et al., 2015; Hall et al., 2015) is compromised by design limitations (e.g. small samples, use of multicomponent interventions and presence of confounders).

An adequately powered randomised trial is needed to address these limitations and reliably quantify the effect of sit-stand desks at work on sitting time, energy expenditure, and cardio-metabolic risk factors in the short- and longer-term (i.e. ≥6 months after desk installation). Prior to conducting this trial, however, there is a need to reduce key uncertainties related to the feasibility and acceptability of the recruitment, measurement, and intervention delivery procedures; these were the objectives of the present study. The specific aims are shown in Box 1.

**Box 1: Study aims.**

1. Assess the feasibility of recruiting eligible participants for the planned trial, by estimating and describing the:
  • proportion of eligible participants expressing trial participation interest;
  • expected recruitment rate;
  • number of recruitment sites needed to achieve the target sample size for the main trial;
  • baseline characteristics of eligible participants with interest in trial participation.

2. Estimate the number of desk mounts (i.e. devices installed on top of conventional workplace desks, which usually include a platform for display units and a work surface and facilitate transitions between sitting and standing, predominantly while performing computer-based activities) and full desks (i.e. desks in which the entire surface area can be adjusted to standing mode) needed for the planned trial, by describing the:
  • proportion of eligible participants preferring desk mounts vs full desks;
  • proportion of eligible participants with workspaces permitting installation of their desk preference.

3. Explore assessment location preferences (home vs workplace vs clinical research facility), to inform the procedures of the planned trial;
4. Assess the feasibility and acceptability of the randomisation;
5. Assess the feasibility and practicalities of intervention delivery;
6. Explore the circumstances under which desks were used in standing mode, to Identify potential barriers that could affect desk use in the planned trial;
7. Estimate retention and attrition rates between baseline and follow-up, to inform the sample size requirements of the planned trial;
8. Explore the acceptability of the intervention, assessments and study procedures assess the variability of outcomes, to inform sample size calculations for the planned trial.

Alt-text: Unlabelled Box

## 2 Methods

### 2.1 Study design

This study assessed the feasibility of conducting a large trial on the use of sit-stand desks at work. The study design and methods are reported elsewhere (Mantzari et al., 2016). Briefly, there were four phases, described in detail below: online survey (Phase I), workspace audit (Phase II), randomised intervention (Phase III), qualitative interviews (Phase IV). It was conducted between May 2015 and December 2016, within two organisations based in Cambridge, England: a genomics company and an NHS Foundation Trust consisting of two hospitals. Ethical approval was obtained from the University of Cambridge Psychology Research Ethics Committee (reference number: PRE.2015.100).

### 2.2 Participant recruitment

Organisation representatives (members of the Occupational Health and Health and Safety teams) circulated a survey link via email along with a brief description of the study to potentially eligible participants, defined as office-based employees who: i) worked at least 60% full-time; ii) spent at least 70% of their working week performing desk-related activities at an organisational workspace; iii) had personal desk allocation; iv) were not already using a sit-stand desk; and v) did not have pre-existing health conditions that made prolonged standing inadvisable.

Of those who completed Phase I, met the eligibility criteria and expressed interest in taking part further, 100 were randomly chosen to complete Phase II. Of those who completed Phase II, had a workspace permitting installation of a sit-stand desk and expressed interest in further participation, 20 participants were randomly chosen to take part in Phase III. Phase IV was completed by those who were randomised to the intervention group in Phase III.

### 2.3 Sample size

As this was a feasibility study to inform the design of a future trial, no formal sample size calculations were produced. The sample sizes for each study phase were pragmatic, based on available resources. They were nonetheless used to determine the precision with which certain parameters can be estimated.

The average participation interest rate reported in previous studies on the use of sit-stand desks is 37%, while the average recruitment rate is 33%. Four-hundred and thirty participants consented to participate in Phase I. Based on this, the 95% confidence intervals around these estimates are between 32% to 42% for interest rates, and 29% and 39% for recruitment rates. The average reported attrition rate between baseline and follow-up is 10% and the maximum attrition rate is 14%. With 20 participants in Phase III, the 95% confidence intervals around these estimates are between 2% and 33% for average attrition and 3.5% and 38% for maximum attrition.

Based on previous studies (Alkhajah et al., 2012; Healy et al., 2013; Neuhaus et al., 2014; Centers for Disease Control, 1999; Speck & Schmitz, 2011; Villars et al., 2012), the expected differences between groups in changes from baseline are: workplace sitting time: −100.77 (17.74) minutes/day; total sitting time: −77 (20.7) minutes/day; workplace standing time: 129.4 (15.8) minutes/day; energy expenditure: 3.58 (12.8) kJ/kg/day. The 95% confidence intervals around these estimates with a sample size of 20 participants in Phase III and 10% attrition are: work sitting time: −108.97 to −92.57; total sitting time: −86.6 to −67.4; work standing time: 122.1 to 136.7; energy expenditure: −2.32 to 9.48.

### 2.4 Phase I procedure

Phase I consisted of an online survey. It included a brief description of the future trial asking participants to indicate their: (a) participation interest in a trial of 3 and 6 months' duration; (b) desk type preferences; and (c) location preferences for baseline and follow-up assessments. At the end of the survey, participants were asked to provide their contact details if they were interested in continuing participation in the study.

### 2.5 Phase II procedure

Phase II involved auditing of 100 workspaces to determine whether and which type of sit-stand desk could be installed. A standardised workspace assessment form was designed to collect information on: (a) desk dimensions; (b) dimensions of available space around the desk; (c) standing transition obstructions; (d) type, number and size of monitors; (e) use of desk-top computer or laptop docking station; (f) presence of desk drawers (attached or detached); and (g) appropriate cable length. On completion of the workspace audit, participants with suitable workspaces were invited to participate in Phase III.

### 2.6 Phase III procedure

Phase III involved 20 participants, 10 of whom were block randomised to the intervention group and 10 to the control group.

### 2.7 Intervention

Participants were provided with a sit-stand desk at work for 3 months.^1^ They were offered one of two desk types –full desk or desk mount- determined by their preference and workspace allowance.

Sit-stand desks were installed after completion of baseline assessments. Full desks (Narbutas electric height-adjustable desk, product code: DHA165) were installed by professionals, after removal of participants' existing desks. Desk mounts (Ergotron WorkFit-TL Desktop Workstation, product code: SKU: 33-406-085) were installed on top of participants' existing desks by a researcher. The researcher also gave a brief demonstration on desk use, along with a leaflet containing information on: i. correct ergonomic posture when standing; ii. Impacts on health of prolonged sitting; iii. How to gradually increase standing time; and iv. How to break up sitting time. Participants allocated to the control group continued to use their existing work desks and were given verbal information on the health impacts of prolonged sitting as well as tips on how to decrease prolonged sitting time at work.

#### 2.7.1 Assessments

Assessments were conducted in workplaces, according to participants' preferences, as assessed in Phase I, at baseline and three-month follow-up and included measurement of standing height, weight and body-fat percentage, waist- and hip-circumference and seated blood pressure. A researcher trained in phlebotomy collected a non-fasting blood sample (in a random subsample of 5 from each group) via venipuncture to measure HbA_1c_, cholesterol and triglycerides.

Participants were also fitted with a combined heart rate and movement monitor (Actiheart), set-up to record with a 15-second epoch, using standard ECG electrodes (3 M™ Red Dot™) on the chest (Brage et al., 2005), and an accelerometer (activPAL3) set up to record acceleration in 20 Hz (Grant et al., 2006), using a nitrile sleeve and waterproof medical grade adhesive dressing (Hypafix® Transparent) on the thigh, both worn 24 h a day, for a 7-day consecutive period. To calibrate heart rate to participants' individual fitness level, a submaximal eight-minute step test was performed at baseline (Brage et al., 2007), allowing estimation of free-living physical activity energy expenditure, which shows good agreement with the Doubly Labelled Water method (Brage et al., 2015). Orientation angle (horizontal or vertical), as well as general movement of the thigh, was determined from thigh acceleration, allowing reliable assessment of the beginning and end of each bout of sitting or lying, standing, and stepping (Grant et al., 2006; Lyden et al., 2012; Kozey-Keadle et al., 2011; Berendsen et al., 2014; Bentley, 2011; Healy et al., 2011). During the 7-day wear period, participants completed a daily log with information on sleep/waking hours, working hours, and any device removal times. During this time, participants also completed a food diary to assess energy intake and questionnaires to assess health- and work-related outcomes (Table 1). Devices, logs and completed questionnaires were collected at the end of the 7-day period. Participants were provided with a personalised report after follow-up assessments, containing information about their physical activity levels, sedentary behaviour and clinical measures.

**Table 1:** Outcomes and measures.

Measure	Outcome and variables
**Phase I**	
Online questionnaire	Interest in trial participation
	Desk type preferences
	Assessment location preferences
	**Sociodemographics** Age Gender BMI Education Occupational role Income

**Phase II**	
	Workspace suitability for full desks and/or desk mounts

**Phase III**	
Delivery time for desks Feasibility of training research staff to install desks Feasibility of trained researcher installing desk mounts Time taken to install each desk Problems associated with desk delivery and installation Feasibility of removing existing desks (applicable only when using full desks)	Practicalities of delivering and implementing intervention
Weekly online diaries	Factors affecting desk use
**Trial-related outcomes**	
	**Behavioural**
Individually calibrated combined heart rate and movement sensing (Actiheart CamNtech Ltd.)	Physical activity energy expenditure^a^
Thigh-worn accelerometer (activPAL PAL Technologies Ltd.)	Sitting time^a^ during (a) working hours; (b) all waking hours
	Standing time^a^ during (a) working hours; (b)all waking hours
	Stepping time^a^ during (a) working hours; (b) all waking hours
	Sitting patterns: (a) Number of sit-to-stand transitions^a^ during (i) working hours; (ii) all waking hours
	(b) Sitting time accrued in prolonged bouts (≥30 min, ≥60 min)^a^, during (i) working hours; (ii) all waking hours
	**Anthropometric and clinical**
Portable stadiometer (Leicester Height Measure Mk II)	Height
Bio-electrical impedance scale (TBF-300A Total Body Composition Analyzer; Tanita)	Weight and body fat percentage
	BMI (kg/m^2^)
Blood pressure monitor (705IT; © OMRON Healthcare Europe B.V.)	Blood pressure
Anthropometric tape measure	Waist and hip circumference
Non-fasting blood tests	Plasma total cholesterol HDL Triglycerides HbA1C
	**Self-report**
Nordic Musculoskeletal Discomfort Questionnaire	Musculoskeletal discomfort
Checklist	Health symptoms (headache, neck pain, fatigue, eye strain, back pain, loss of concentration)
Work Ability Index	Ability to work
Work Performance and Health Questionnaire	Work productivity
The Stanford Presenteeism Scale	Presenteeism and absenteeism
Brief Job Satisfaction Measure II	Job satisfaction
SIT-Q-7d	Domain-specific sedentary behaviour
Euro-Quality of Life 5	Health-related quality of life
4-day estimated food diary	Food and drink intake to assess potential compensation effects in terms of energy intake

**Phase IV**	
Qualitative interviews	Acceptability of intervention
	Acceptability of assessments and burden
	Acceptability of study procedures

a Outcomes normalized to an 8-hour workday or a 16-hour waking day, to account for variations in work or waking time schedules and monitor wear time.

Participants allocated to the intervention group also completed an online diary once a week, for the duration of the study. This involved responding to structured questions about each hour of the working day whether they were sitting, standing, or were away from their desk, the types of tasks they carried out and their reasons for switching from standing-to-sitting and vice versa (Appendix-Text S2; Figs. S1 &S2; Tables S2 and S3).

### 2.8 Phase IV procedure

At the end of Phase III, participants allocated to the intervention group were interviewed about their experiences of taking part in the study. Interviews were semi-structured and lasted approximately 20 min.

### 2.9 Outcomes and measures

The measures included in each phase of the study are presented Table 1.

### 2.10 Data processing

Heart rate data from the Actiheart monitors were pre-processed to reduce potential noise using the Bayesian approach by Stegle et al. (2008). Accelerometry data were checked for anomalies. Non-wear periods, defined as periods >90 min of zero acceleration, accompanied by non-physiological heart rate data, were excluded from the analyses. For each participant, minute-to-minute estimates of Physical Activity Energy Expenditure (PAEE) in kJ/kg were derived by heart rate and accelerometry measures using a “branched equation model” (Brage et al., 2004). The relationship between heart rate and PAEE was calibrated using data from the individually performed submaximal step test. From the PAEE estimates, the fraction of time (per hour) spent in PA intensity groups, expressed as multiples of predicted resting metabolic rate (METs), was derived and then converted to time spent in minutes per 24 h day. All measures were summarised to daily measures. Only measures from participants with a minimum Actiheart wear time of 80% per 24 h day were considered valid for analysis.

ActivPAL events files were processed in SAS 9.4. For each participant, the time spent sitting, standing, stepping and sitting in bouts lasting between 10 and 180 min and the number of sit-stand transitions were totaled for each day for all waking hours and all work hours. To be included in the analyses, participants needed to have worn the monitor for ≥2 working days and ≥1 non-working day at both baseline and follow-up. Each activity was then averaged across valid workdays and non-workdays separately and across all days overall. Valid days were classified as those during which the monitor was worn for ≥10 waking hours and ≥75% of working hours.

### 2.11 Data analysis

Descriptive statistics are reported for feasibility and acceptability outcomes. To estimate potential effect sizes, between group differences were computed in average change from baseline in energy expenditure and sitting time during working and non-working days, as well as in standing time, stepping time, number of sit-to-stand transitions, time spent in prolonged bouts of sitting (>/30 min and >/60 min), time spent in moderate physical activity (MPA; defined as activity in 3–6 METs) and time spent in vigorous physical activity (VPA; defined as activity in >6 METs).

## 3 Results

### 3.1 Phase I

#### 3.1.1 Feasibility of recruiting eligible participants

**Fig. 1:**
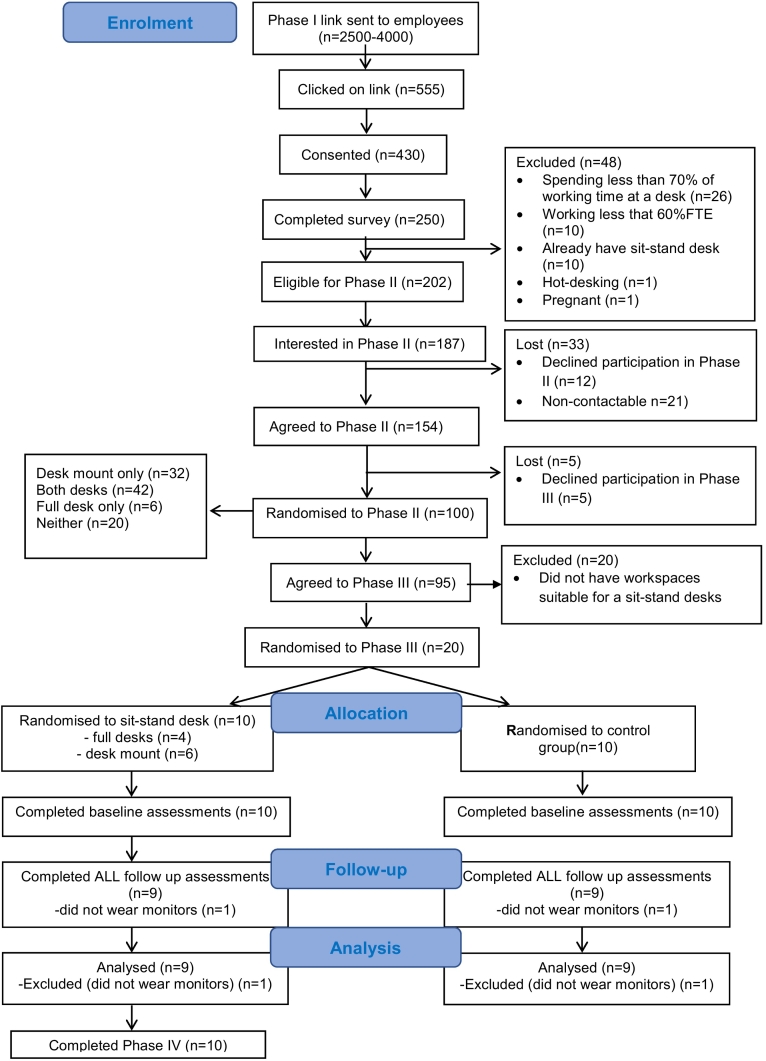
Flow of participants through the study.

Ninety-two percent (187/202) of eligible respondents expressed participation interest for the future trial.^2^ The survey response rate was between 14% and 23% (555 of 2600–4000 employees on the email list (exact size unknown) clicked on the survey link in a non-personalised, circular email). The completion rate was between 5% and 7.5% (Fig. 1).

Among survey completers, 81% were eligible for the proposed trial and therefore considered for Phase II. The recruitment rate to Phase III among those consenting to Phase I was 33% (95/430).

#### 3.1.2 Characteristics of eligible participants

Most respondents were women, working full-time in professional job roles or positions involving clerical and administrative support. Just over half (55%) were highly educated, having completed undergraduate and postgraduate university degrees, and had a high income (i.e. £ > 25 k) (55%). Over half were overweight or obese (54%). The characteristics of those completing Phases II and III were similar (Table 2). Information regarding the representativeness of the sample can be found in the Appendix – Text S1.

**Table 2:** Characteristics of survey completers (Phase I) who were eligible for the main trial (*n* = 187).

**Sex**	
Men	39%
Women	61%
**Age in years** (Mean (sd))	43.4 (11.2)
**BMI in kg/m**^**2**^ >25 (healthy) 25–30 (overweight) >30 (obese) Mean (sd)	46% 28% 26% 26.7 (6.82)
**Occupational role**	
Executive, administrator, or senior manager	15%
Professional	41%
Technical support	2%
Sales	0%
Clerical and administrative support	41%
Service occupation	1%
**% FTE**	
Full time	86%
Part time	14%
**Number of working hours/week** (Mean (sd)	36.8 (5.47)
**Number of hours spent at desk/day (**Mean (sd))	7 (1.65)
**Education**	
Less than A Levels (no formal educational qualifications or O Levels/GCSEs or equivalent) A Levels or equivalent	12.5% 12.5%
Vocational education	11%
Completed undergraduate degree	24%
Completed post-graduate degree	31%
Other	9%
**Annual income**	
Under £15,000	3%
£15–25,000	33%
£25–35,000	25%
£35–£50,000	22%
£50–£70,000	5%
Above £70,000	3%
Prefer not to say	9%

#### 3.1.3 Preferences for desk-type and assessment location

Among respondents, 38% preferred a full-desk, 24% a desk-mount and 38% expressed no preference; 71% preferred to be assessed in the workplace, 17% in a laboratory facility, 4% at home and 24% expressed no preference.

### 3.2 Phase II

Forty-two percent of assessed workspaces were suitable for installation of either full desks or desk mounts, 32% were only suitable for a desk mount, 6% for a full desk and 20% could not accommodate any sit-stand desk type, predominantly due to a lack of space for putting the desk into standing mode. Sixty-eight percent of workspaces allowed for installation of participants' preferred desk type.

### 3.3 Phase III

#### 3.3.1 Feasibility of trial procedures and intervention delivery

No major problems were reported with the study procedures, including intervention delivery. Randomisation was feasible and acceptable, generating trial groups with similar characteristics. Of those randomised to the intervention group, four received full desks and six received desk mounts; all were installed with minimal disruption to participants' work.

Conducting assessments in the workplace was feasible, including venipuncture, which was successfully undertaken for 90% of participants by a researcher trained in phlebotomy. One participant refused repeat venipuncture after an initial failed attempt. All participants agreed to wear both activity monitors at baseline and no major issues were reported with their fitting or use. Technical faults with recording of the combined sensor occurred during the baseline assessments of the first three participants to complete these. These faults resulted from issues related to charging of the device but provided enough data for affected participants to be included in analyses. It was feasible to use all the pre-specified questionnaires to assess self-reported work and health-related outcomes.

#### 3.3.2 Participant retention

Among participants who completed Phase II, 5% declined participation in Phase III. Of those randomised in Phase III, attrition between consenting to take part and the baseline assessments was 5% (1/20). No participants dropped out between baseline and follow-up; however, two participants (10% of those randomised, one in each group) opted out of wearing both the thigh and chest monitors at follow up, due to perceived inconvenience.

#### 3.3.3 Changes in energy expenditure, sitting time, anthropometric and clinical outcomes, standing, stepping, sitting patterns and activity patterns

Compared to the control group, the use of sit-stand desks reduced sitting time, at work by 94 min (95%CI: (−170.7 to −17.7) per 8-h working day (Table 3). No clear trend emerged with regards to the impact of sit-stand desks on energy expenditure.

**Table 3:** Mean (SD) energy expenditure (PAEE in kj/kg/day), sitting time (minutes) anthropometric and blood-related values at baseline and follow-up according to group.

Measure	Intervention (*n* = 9)		Control (n = 9)		Intervention- Control
	Baseline	Follow-up	Baseline	Follow-up	Difference in change from baseline (95% CI)
**PAEE (kj/kg/day)**					
Waking hrs, all days	55.1 (16.9)	55.4 (20.4)	43.6 (16.9)	43.9 (11.4)	0.14 (−9.73, 10.0)
Waking hrs, working days	53.4 (21.3)	52.6 (18.3)	38.5 (15.0)	42.0 (16.8)	−4.3 (−17.6, 9.0)
Working hrs, working days	18.1 (10.7)	18.8 (7.1)	14.2 (9.2)	12.9 (5.0)	1.95 (−6.29, 10.1)
Waking hrs, non-working	58.4 (24.4)	60.5 (24.8)	44.3 (18.2)	50.9 (15.6)	−4.54 (−18.7, 9.6)
**Sitting time (min)**					
Waking hrs, all days	627.1 (52.4)	583.4 (108.3)	620.9 (67.0)	637.1 (53.2)	−59.9 (−125.2, 5.5)
Waking hrs, working days	659.5 (73.1)	625.9 (160.0)	676.9 (75.8)	729.8 (91.2)	−86.5 (−250.4, 77.4)
Working hrs, working days	379.9 (57.7)	301.3 (104.5)	387.1 (31.1)	402.7 (23.5)	−94.2 (−170.7, −17.7)
Waking hrs, non-working	572.2 (87.8)	563.0 (153.8)	535.9(114.4)	519.1 (60.6)	9.73 (−94.4, 113.9)
**Systolic Blood Pressure**	125.5 (10.1)	126.4 (13.6)	129.7 (14.6)	128.4 (13.2)	2.15 (5.99, −10.3)
**Diastolic Blood Pressure**	76.1 (7.14)	79.6 (7.29)	79.6 (11.1)	77.9 (10.3)	5.25 (1.13, 9.37)
**Heart rate**	73.5 (14.9)	72.4 (13.1)	67.4 (6.6)	64.9 (7.4)	1.15 (−10.5, 12.8,)
**Waist circumference**	91.6 (18.0)	92.0 (19.0)	83.8 (6.5)	82.4 (5.8)	1.81 (−1.11, 4.74)
**Hip circumference**	107.6 (11.6)	107.2 (12.3)	99.8 (4.6)	99.0 (4.6)	0.35 (−1.39, 2.11)
**Weight**	78.9 (17.9)	79.5 (17.5)	70.1 (10.6)	70.0 (10.5)	0.91 (−0.91, 2.73)
**BMI**	27.0 (6.18)	27.3 (6.35)	23.1 (2.9)	23.1 (2.9)	0.34 (−0.37, 1.05)
**Body fat percentage**	29.4 (11.9)	29.0 (12.0)	24.1 (8.7)	23.2 (8.1)	0.45 (−1.46, 2.36)
**HbA1c**^a^	32.7 (2.06)	33.5 (2.89)	33.2 (3.9)	31.2 (3.2)	2.75 (−0.31, 5.81)
**Cholesterol**^a^	4.97 (1.13)	4.90)0.91)	4.65 (0.91)	4.37 (1.22)	0.20 (−0.39, 0.79)
**HDL**^a^	1.56 (0.35)	1.69 (0.45)	1.33 (0.28)	1.41 (0.24)	0.06 (−0.29, 0.41)
**LDL**^a^	2.81 (1.29)	2.66 (1.07)	2.68 (0.60)	2.56 (1.16)	0.35 (−0.55, 1.25)
**Triglycerides**^a^	1.32 (0.05)	1.17 (0.260	1.40 (0.89)	0.90 (0.24)	0.35 (−0.55, 1.25)

a Blood-related outcomes were assessed in 10 participants in total (5 in the intervention and 5 in the control group).

The findings suggest a possible trend in favour of the control group for all anthropometric and clinical outcomes (Table 3). The results also suggest that the use of sit-stand desks might have: i) increased standing time during waking hours of working days (60.7 min (−12.1, 133.5); ii) decreased prolonged sitting time during waking hours of working days and during working hours (waking hours: −120.6 min (−355.2, 113.9); working hours: −100.2 min (−318.5, 118.0); and iii) increased prolonged sitting time during non-working days, (>/30 min bouts: 249.6 min (−244.2, 743.4); >/60 min bouts: 241.1 min (−149.3, 631.5)) (Appendix, Table S1).

#### 3.3.4 Factors affecting sit-stand desk use

The factors and circumstances affecting sit-stand desk use, as assessed by weekly online diaries, are presented in the Appendix-Text S2.

### 3.4 Phase IV

#### 3.4.1 Acceptability of the study procedures and assessments

The study procedures and intervention were considered acceptable. Participants expressed positive attitudes towards the study and the sit-stand desks. Assessments were generally considered acceptable. Some participants reported challenges associated with using the monitors, which in a minority of cases rendered them unacceptable. Completing the food questionnaire and online activity diary were considered challenging (Quotes - Appendix Table S4).

## 4 Discussion

This multiphase study demonstrated the feasibility and acceptability of conducting a trial to evaluate the impact of providing sit-stand desks at work on overall energy expenditure, sitting time, and cardio-metabolic risk factors, in the short- and longer-term. It was feasible to identify, recruit and retain eligible participants, estimate desk type and measurement location preferences, and assess the suitability of workspaces for sit-stand desk installation. This study informs the design of the main trial and suggests that the proposed procedures, including randomisation, intervention delivery and assessments, are feasible and acceptable.

The present study demonstrated the feasibility of delivering an intervention to participants, which, consistent with previous research (Alkhajah et al., 2012; Dutta et al., 2014; Neuhaus et al., 2014; DM et al., 2014), reduced their sitting time at work. The planned trial is expected to be one of the largest known trials of the use of sit-stand desks at work, aiming to recruit 500 participants. One of the key uncertainties related to its design was the feasibility of recruiting the required sample. The present feasibility study demonstrated high levels of participation interest among eligible participants and provided estimates of expected recruitment rates, which fall within typical recruitment rates for prevention trials (Cooper et al., 2015). Based on the findings, it is estimated that to recruit the target sample, approximately 10,000 office based employees need to be approached. This would require approaching approximately seven large UK companies (defined as those with 250+ employees), given that the average number of employees of such companies is approximately 1500 (Rhodes, 2016). The study also helped identify strategies to maximise recruitment, i.e. by involving organisation representatives and team managers into the recruitment process. Differences were observed in the way the two participating organisations promoted the study among their employees, which resulted in correspondingly different response rates. Representatives of the genomics company informed their employees of the study, prior to them receiving the circular email. The response rate from this organisation was higher than that from the hospital employees, who did not receive any prior information about the study.

Another key uncertainty of the planned trial was the feasibility of randomising participants at the individual level; some existing trials of sit-stand desks at work have employed cluster randomised designs, in which sites, rather than individuals, have been randomised (Clemes et al., 2014; Buman et al., 2017; Healy et al., 2016; Danquah et al., 2017). Whilst this approach avoids differential treatment of employees within the same organisation and potential contamination between intervention and control participants, such designs result in reduced statistical power and hence require larger sample sizes, and potential recruitment bias (Torgerson, 2001). Given the already large sample of the proposed trial, increasing it further is unlikely to impact feasibility; moreover, there was no evidence of contamination i.e. control participants using sit-stand desks, thus demonstrating the feasibility, acceptability and success of randomising participants at the individual level. The study also demonstrated the feasibility of conducting all the assessments in workplaces, which the majority of participants preferred. This has important implications for future studies; the assessments of the planned trial include several measurements, which are traditionally conducted in clinical research facilities. It was demonstrated here that they can be done in the recruited workplaces, with appropriate training of the research team and liaison with facilities managers.

A further uncertainty of the proposed trial was the required number of each desk type. Full desks have been generally considered superior to desk mounts, due to stability and working space (Chau et al., 2014) and their use is recommended (Neuhaus et al., 2014). However, the results of this study demonstrate that not all office-based employees agree. Although most participants preferred either full desks or had no preference, approximately a quarter preferred a desk mount. This highlights the need to take participant preferences into account in determining appropriate desk types, in order to try and maximise the probability of desk-use. Based on participants' desk type preferences and the workspace assessments, we estimated that approximately 50% of the desks needed for the main trial will be full desks. However, not all workspaces allow for installation of both types of desks and importantly, many are not suitable for receiving any type of sit-stand desk, an issue also not previously considered.

One of the key uncertainties related to the provision of sit-stand desks at work is their potential for compensation effects and therefore their impact on cardio-metabolic health (MacEwen et al., 2015). Our findings highlight concerns that widespread adoption of sit-stand desks may not decrease the risk of cardio-metabolic disease. Caution is warranted in interpreting the study findings as it was not powered to detect effects. However, results highlight the possibility that use of sit-stand desks may result in increased levels of prolonged sitting time during non-working days, and adverse anthropometric and clinical outcomes. These observed trends underline the need to clarify the uncertainties surrounding sit-stand desks' potential health benefits and harms. The full trial should do this by focusing on the impact on energy expenditure in and outside of work, as well as short and longer term (i.e. 6 months after desk installation) sitting time and cardio-metabolic risk factors, using a rigorous design and robust measures.

In conclusion, the findings of the present study support the feasibility and acceptability of conducting a large randomised controlled trial to assess the impact of sit-stand desks at work on energy expenditure, sitting time and cardio-metabolic risk. Preliminary evidence suggests the desks' potential to reduce workplace sitting but raises concern about their potential to adversely affect energy expenditure and sitting time outside work as well as cardio-metabolic risk factors, thus highlighting the need for further research into their potential behavioural compensation and overall health impact.

## Funding

This report is independent research funded by the National Institute for Health Research Policy Research Programme (Policy Research Unit in Behaviour and Health [PR-UN-0409-10109]), the Medical Research Council [Unit Programme number MC_UU_12015/3] and the British Heart Foundation [Intermediate Basic Science Research Fellowship grant FS/12/58/29709 to KW]. The views expressed in this publication are those of the authors and not necessarily those of the NHS, the National Institute for Health Research, the Department of Health and Social Care or its arm's length bodies, and other Government Departments, the Medical Research Council, or the British Heart Foundation. The final version of the report and ultimate decision to submit for publication was determined by the authors.

## Competing interests

Eleni Mantzari, Catherine Galloway, Katrien Wijndaele, Soren Brage, Simon Griffin and Theresa M Marteau all have no financial disclosures and declare that they have no competing interests.

## Appendix A Supplementary data

Supplementary material Image 1

## Footnotes

1 In the planned trial, participants will be provided with desks for 6 months, in order to allow for assessment of long-term effects. Due to limited resources in this feasibility study, the duration of the intervention was reduced to 3 months. To assess whether there would be differences in participation interest as a result of the different study durations, in Phase I, participants were asked about their willingness to participate in both a 3-month and a 6-month trial.

2 Results refer to interest in participating in a 6-month trial

## References

[bb0005] Adeleke S.O., Healy G.N., Smith C., Goode A.D., Clark B.K. (2017). Effect of a workplace-driven sit–stand initiative on sitting time and work outcomes. Translational Journal of the American College of Sports Medicine.

[bb0010] Alkhajah T.A., Reeves M.M., Eakin E.G., Winkler E.A., Owen N., Healy G.N. (2012). Sit–stand workstations: a pilot intervention to reduce office sitting time. Am. J. Prev. Med..

[bb0015] Bentley I. (2011). Comparison of the Accuracy of the ActivPAL Activity Monitor, the Actigraph GT1M Activity Monitor and the Actigraph GT3X Activity Monitor During Activities Of Daily Living.

[bb0020] Berendsen B., Hendriks M., Meijer K., Plasqui G., Schaper N., Savelberg H. (2014). Which activity monitor to use? Validity, reproducibility and user friendliness of three activity monitors. BMC Public Health.

[bb0025] Biswas A., Oh P.I., Faulkner G.E. (2015). Sedentary time and its association with risk for disease incidence, mortality, and hospitalization in adults: a systematic review and meta-analysis sedentary time and disease incidence, mortality, and hospitalization. Ann. Intern. Med..

[bb0030] Brage S., Brage N., Franks P.W. (2004). Branched equation modeling of simultaneous accelerometry and heart rate monitoring improves estimate of directly measured physical activity energy expenditure. J. Appl. Physiol..

[bb0035] Brage S., Brage N., Franks P., Ekelund U., Wareham N. (2005). Reliability and validity of the combined heart rate and movement sensor Actiheart. Eur. J. Clin. Nutr..

[bb0040] Brage S., Ekelund U., Brage N. (2007). Hierarchy of individual calibration levels for heart rate and accelerometry to measure physical activity. J. Appl. Physiol..

[bb0045] Brage S., Westgate K., Franks P.W. (2015). Estimation of free-living energy expenditure by heart rate and movement sensing: a doubly-labelled water study. PLoS One.

[bb0050] Brocklebank L.A., Falconer C.L., Page A.S., Perry R., Cooper A.R. (2015). Accelerometer-measured sedentary time and cardiometabolic biomarkers: a systematic review. Prev. Med..

[bb0055] Buman M.P., Mullane S.L., Toledo M.J. (2017). An intervention to reduce sitting and increase light-intensity physical activity at work: design and rationale of the ‘Stand & Move at Work’ group randomized trial. Contemp. Clin. Trials.

[bb0060] Centers for Disease Control (1999). Promoting physical activity: a guide for community action: human kinetics.

[bb0065] Chastin S.F., Egerton T., Leask C., Stamatakis E. (2015). Meta-analysis of the relationship between breaks in sedentary behavior and cardiometabolic health. Obesity.

[bb0070] Chau J.Y., Grunseit A.C., Chey T. (2013). Daily sitting time and all-cause mortality: a meta-analysis. PLoS One.

[bb0075] Chau J., Daley M., Srinivasan A., Dunn S., Bauman A., van der Ploeg H. (2014). Desk-based workers' perspectives on using sit-stand workstations: a qualitative analysis of the Stand@Work study. BMC Public Health.

[bb0080] Chau J.Y., Sukala W., Fedel K. (2016). More standing and just as productive: effects of a sit-stand desk intervention on call center workers' sitting, standing, and productivity at work in the Opt to Stand pilot study. Prev. Med. Rep..

[bb0085] Clemes S.A., O'Connell S.E., Edwardson C.L. (2014). Office workers' objectively measured sedentary behavior and physical activity during and outside working hours. J. Occup. Environ. Med..

[bb0090] Cooper C.L., Hind D., Duncan R. (2015). A rapid review indicated higher recruitment rates in treatment trials than in prevention trials. J. Clin. Epidemiol..

[bb0095] Danquah I.H., Kloster S., Holtermann A., Aadahl M., Tolstrup J.S. (2017). Effects on musculoskeletal pain from “Take a Stand!”–a cluster-randomized controlled trial reducing sitting time among office workers. Scand. J. Work Environ. Health.

[bb0100] DM Chau J.Y., Dunn S., Srinivasan A., Do A., Bauman A.E., van der Ploeg H.P. (2014). The effectiveness of sit-stand workstations for changing office workers' sitting time: results from the Stand@Work randomized controlled trial. Int. J. Behav. Nutr. Phys. Act..

[bb0105] Dutta N., Koepp G.A., Stovitz S.D., Levine J.A., Pereira M.A. (2014). Using sit-stand workstations to decrease sedentary time in office workers: a randomized crossover trial. Int. J. Environ. Res. Public Health.

[bb0110] Ekelund U., Steene-Johannessen J., Brown W.J. (2016). Does physical activity attenuate, or even eliminate, the detrimental association of sitting time with mortality? A harmonised meta-analysis of data from more than 1 million men and women. Lancet.

[bb0115] Ellegast R., Weber B., Mahlberg R. (2012). Method inventory for assessment of physical activity at VDU workplaces. Work.

[bb0120] Grant P.M., Ryan C.G., Tigbe W.W., Granat M.H. (2006). The validation of a novel activity monitor in the measurement of posture and motion during everyday activities. Br. J. Sports Med..

[bb0125] Graves L., Murphy R., Shepherd S.O., Cabot J., Hopkins N.D. (2015). Evaluation of sit-stand workstations in an office setting: a randomised controlled trial. BMC Public Health.

[bb0130] Hall J., Mansfield L., Kay T., McConnell A.L. (2015). The effect of a sit-stand workstation intervention on daily sitting, standing and physical activity: protocol for a 12 month workplace randomised control trial. BMC Public Health.

[bb0135] Healy G., Anuradha S., Osman A. (2011). Comparison of the GT3X-plus and activPAL monitors in controlled and free-living environments: Accuracy and responsiveness to change. Paper Presented at: 2011 Annual Meeting of the International Society for Behavioral Nutrition and Physical Activity.

[bb0140] Healy G., Lawler S., Thorp A. (2012). Reducing prolonged sitting in the workplace. Human Factors and Ergonomics Society.

[bb0145] Healy G.N., Eakin E.G., Lamontagne A.D. (2013). Reducing sitting time in office workers: short-term efficacy of a multicomponent intervention. Prev. Med..

[bb0150] Healy G.N., Eakin E.G., Owen N. (2016). A cluster RCT to reduce office Workers' sitting time: Impact on activity outcomes. Med. Sci. Sports Exerc..

[bb0155] Hedge A. (2004). Effects of an electric height-adjustable worksurface on self-assessed musculoskeletal discomfort and productivity in computer workers. Methods.

[bb0160] Kozey-Keadle S., Libertine A., Lyden K., Staudenmayer J., Freedson P.S. (2011). Validation of wearable monitors for assessing sedentary behavior. Med. Sci. Sports Exerc..

[bb0165] Lee I.-M., Shiroma E.J., Lobelo F., Puska P., Blair S.N., Katzmarzyk P.T. (2012). Effect of physical inactivity on major non-communicable diseases worldwide: an analysis of burden of disease and life expectancy. Lancet.

[bb0170] Lyden K., Kozey-Keadle S.L., Staudenmayer J.W., Freedson P.S. (2012). Validity of two wearable monitors to estimate breaks from sedentary time. Med. Sci. Sports Exerc..

[bb0175] MacEwen B.T., MacDonald D.J., Burr J.F. (2015). A systematic review of standing and treadmill desks in the workplace. Prev. Med..

[bb0180] Mansoubi M., Pearson N., Biddle S.J., Clemes S.A. (2015). using sit-to-stand workstations in offices: is there a compensation effect?. Med. Sci. Sports Exerc..

[bb0185] Mantzari E., Wijndaele K., Brage S., Griffin S.J., Marteau T.M. (2016). Impact of sit-stand desks at work on energy expenditure and sedentary time: protocol for a feasibility study. Pilot and Feasibility Studies.

[bb0190] Neuhaus M., Healy G.N., Dunstan D.W., Owen N., Eakin E.G. (2014). Workplace sitting and height-adjustable workstations: a randomized controlled trial. Am. J. Prev. Med..

[bb0195] Ng S.W., Popkin B. (2012). Time use and physical activity: a shift away from movement across the globe. Obes. Rev..

[bb0200] O'Connell S., Jackson B., Edwardson C. (2015). Providing NHS staff with height-adjustable workstations and behaviour change strategies to reduce workplace sitting time: protocol for the Stand More AT (SMArT) work cluster randomised controlled trial. BMC Public Health.

[bb0205] Offıce for National Statistics (2011). Labour force survey: employment status by occupation.

[bb0210] Parry S., Straker L. (2013). The contribution of office work to sedentary behaviour associated risk. BMC Public Health.

[bb0215] Peddie M.C., Bone J.L., Rehrer N.J., Skeaff C.M., Gray A.R., Perry T.L. (2013). Breaking prolonged sitting reduces postprandial glycemia in healthy, normal-weight adults: a randomized crossover trial. Am. J. Clin. Nutr..

[bb0220] Radas A., Mackey M., Leaver A. (2013). Evaluation of ergonomic and education interventions to reduce occupational sitting in office-based university workers: study protocol for a randomized controlled trial. Trials.

[bb0225] Rhodes C. (2016). Business Statistics.

[bb0230] Sandy M.E. (2016). Longitudinal Study of Adjustable Workstations.

[bb0235] Schwartz B., Kapellusch J.M., Schrempf A., Probst K., Haller M., Baca A. (2016). Effect of a novel two-desk sit-to-stand workplace (ACTIVE OFFICE) on sitting time, performance and physiological parameters: protocol for a randomized control trial. BMC Public Health.

[bb0240] Speck R.M., Schmitz K.H. (2011). Energy expenditure comparison: a pilot study of standing instead of sitting at work for obesity prevention. Prev. Med..

[bb0245] Stegle O., Fallert S.V., MacKay D.J., Brage S. (2008). Gaussian process robust regression for noisy heart rate data. IEEE Trans. Biomed. Eng..

[bb0250] Straker L., Abbott R.A., Heiden M., Mathiassen S.E., Toomingas A. (2013). Sit–stand desks in call centres: associations of use and ergonomics awareness with sedentary behavior. Appl. Ergon..

[bb0255] Tobin R., Leavy J., Jancey J. (2016). Uprising: an examination of sit-stand workstations, mental health and work ability in sedentary office workers, in Western Australia. Work.

[bb0260] Torgerson D.J. (2001). Contamination in trials: is cluster randomisation the answer?. BMJ [Br. Med. J.].

[bb0265] Tremblay M.S., Aubert S., Barnes J.D. (2017). Sedentary Behavior Research Network (SBRN)–terminology consensus project process and outcome. Int. J. Behav. Nutr. Phys. Act..

[bb0270] Villars C., Bergouignan A., Dugas J. (2012). Validity of combining heart rate and uniaxial acceleration to measure free-living physical activity energy expenditure in young men. J. Appl. Physiol..

[bb0275] Wilmot E., Edwardson C., Achana F. (2012). Sedentary time in adults and the association with diabetes, cardiovascular disease and death: systematic review and meta-analysis. Diabetologia.

