# Supplementary material for: Impact of sit-stand desks at work on energy expenditure, sitting time and cardio-metabolic risk factors: Multiphase feasibility study with randomised controlled component
Source: Prev Med Rep. 2018 Nov 26;13:64–72. doi: 10.1016/j.pmedr.2018.11.012 (PMC6603239; doi:10.1016/j.pmedr.2018.11.012)
Supplement: Supplementary file 1 — Supplementary material [file mmc1.docx]

**APPENDIX - Supplementary material**

**Text S1: Sample representativeness**

Ninety-one percent of the survey (Phase I) respondents were NHS staff. Consistent with this, the sample (the characteristics of which are displayed in Table 2 of the main manuscript) is comparable to the NHS workforce, of whom the majority is female^^[[1]](#footnote-1)^^, 60% are classified as overweight and obese^^[[2]](#footnote-2)^^, 54% are professionally qualified and 46% constitute support staff^^[[3]](#footnote-3)^^, and 89% work full time and have a mean age of 42 years^^[[4]](#footnote-4)^^ and a mean income of around £31,000^^[[5]](#footnote-5)^^. As NHS is one of the largest employers in the world, and the largest in Europe, with over 1.6 million employees^^[[6]](#footnote-6)^^, findings generated based on NHS staff are potentially generalisable to the largest employee group in the UK. It is also worth noting that in terms BMI, the sample is also comparable to other UK employees^^[[7]](#footnote-7)^^, while overweight and obesity rates are representative of the general population, 62% of which is classified as overweight and obese^^[[8]](#footnote-8)^^ (HSE 2014). These data suggest that respondents were representative of the population from which they were selected while findings are potentially generalisable to UK employees more widely.

**Table S1:** Mean (sd) standing (minutes), stepping (minutes), prolonged sitting (minutes), sit-stand transitions (number per day), time spent in MPA (minutes) at baseline and follow-up according to day type and group

|  | **Measure** | **Intervention (n=9)** | | **Control (n=9)** | | **Intervention-Control** |
| --- | --- | --- | --- | --- | --- | --- |
|  |  | **Baseline** | **Follow-up** | **Baseline** | **Follow-up** | **Difference in change**  **from baseline (95% CI)** |
| **Waking hrs,**  **all days** | **Standing time (min)** | 206.4 (35.8) | 253.5 (86.5) | 235.5 (54.2) | 220.9 (39.6) | 61.6 (-0.37, 123.6) |
|  | **Stepping time (min)** | 126.5 (29.2) | 123.1 (34.9) | 103.5 (26.1) | 101.9 (18.9) | -1.77 (-23.9, 20.3) |
|  | **Prolonged sitting (>=30minutes) (min)** | 598.6 (211.7) | 607.8 (341.6) | 677.0 (308.5) | 674.5 (241.1) | 11.73 (-174.1, 197.51) |
|  | **Prolonged sitting (>=60minutes) (min)** | 253.9 (143.2) | 307.8 (246.6) | 329.7 (225.9) | 314.9 (171.3) | 68.6 (-71.5, 208.8) |
|  | **Number of sit-stand transitions** | 55.1 (6.7) | 54.3 (9.7) | 53.4(10.9) | 51.8 (8.7) | -3.09 (-5.72, 738) |
|  | **Time spent in MPA (min)** | 87.7 (35.1) | 83.4 (47.0) | 52.9 (34.9) | 56.1 (21.5) | -8.29 (-35.3, 18.7) |
|  | **Time spent in VPA (min)** | 21.0 (17.6) | 18.8 (18.7) | 7.0 (10.6) | 9.2 (9.3) | -2.34 (-10.1, 5.4) |
| **Waking hrs, working days** | **Standing time (min** | 185.8 (62.7) | 233.6 (120.4) | 190.8 (60.5) | 177.8 (27.6) | 60.7 (-12.1, 133.5) |
|  | **Stepping time (min)** | 114.6 (31.8) | 118.5 (30.9) | 92.4 (29.6) | 91.9 (24.5) | 4.39 (-24.4, 33.2) |
|  | **Prolonged sitting (>=30minutes) (min)** | 601.6 (280.0) | 511.5 (309.7) | 741.6 (344.7) | 771.6 (289.4) | -120.6 (-355.2, 113.9) |
|  | **Prolonged sitting (>=60minutes) (min)** | 244.0 (173.3) | 236.6 (208.1) | 352.4 (266.7) | 347.1 (210.1) | -2.13 (-202.2, 197.9) |
|  | **Number of sit-stand transitions** | 55.8 (9.6) | 55.3 (12.8) | 56.6 (9.8) | 46.3 (8.0) | 3.7 (-2.7, 10.3) |
|  | **Time spent in MPA (min)** | 53.4 (21.3) | 52.6 (18.3) | 38.5 (15.0) | 42.0 (16.8) | -4.3 (-17.6, 9.0) |
|  | **Time spent in VPA (min)** | 18.1 (10.7) | 18.8 (7.1) | 14.2 (9.2) | 12.9 (5.0) | 1.95 (-6.29, 10.1) |
| **Working hrs, working days** | **Standing time (min** | 17.8 (6.4) | 33.6 (13.8) | 18.7 (5.6) | 32.4 (10.0) | -2.12 (-8.64, 12.9) |
|  | **Stepping time (min)** | 26.9 (8.8) | 23.9 (12.7) | 23.0 (9.7) | 18.8 (5.9) | 1.21 (-4.79, 7.21) |
|  | **Prolonged sitting (>=30minutes) (min** | 338.6 (209.2) | 263.0 (235.4) | 496.7 (368.4) | 521.3 (255.4) | -100.2 (-318.5, 118.0) |
|  | **Prolonged sitting (>=60minutes) (min)** | 125.7 (121.4) | 109.4 (146.7) | 253.4 (267.5) | 238.9 (180.3) | -1.77 (-179.8, 176.2) |
|  | **Number of sit-stand transitions** | 65.4 (51.2) | 145.1 (103.2) | 56.9 (27.9) | 44.9 (18.6) | 91.7 (15.3, 168.0) |
|  | **Time spent in MPA (min)** | N/A | | | | |
|  | **Time spent in VPA (min)** | N/A | | | | |
| **Waking hrs, non-working days** | **Standing time (min** | 243.7 (59.9) | 256.3 (98.1) | 305.2 (97.2) | 316.0 (49.9) | 3.35 (-88.1, 94.8) |
|  | **Stepping time (min)** | 144.0 (39.3) | 140.7 (198.3) | 118.9 (35.3) | 124.9 (22.3) | -13.1 (-53.8, 27.6) |
|  | **Prolonged sitting (>=30minutes) (min)** | 559.8 (198.3) | 666.7 (518.3) | 572.3 (557.0) | 419.2 (195.5) | 249.6 (-244.2, 743.4) |
|  | **Prolonged sitting (>=60minutes) (min)** | 244.1 (111.8) | 398.4 (418.7) | 295.1 (394.1) | 201.1 (148.2) | 241,1 (-149.3, 631.5) |
|  | **Number of sit-stand transitions** | 54.8 (9.8) | 54.1 (13.6) | 60.3 (20.2) | 63.3 (11.8) | -1.94 (-16.5, 12.6) |
|  | **Time spent in MPA (min)** | 69.5 (44.1) | 73.8 (41.6) | 85.2 (56.9) | 82.8 (48.7) | 1.34 (-53.7, 56.3) |
|  | **Time spent in VPA (min)** | 12.1 (28.1) | 12.5 (21.5) | 9.3 (10.8) | 10.0 (16.9) | -1.59 (-11.85, 8.68) |

Outcomes standardised to 8 hour work days and 16 hour waking days

**Text S2: Factors affecting sit-stand desk use**

The possible factors affecting sit-stand desk use were explored through the online diaries each intervention participant was requested to fill once each week. All participants adhered to this request. Specifically, once a week, participants were sent a link to the their online diary and asked to indicate for each working hour of that day whether they were sitting, standing or away from their desk, what type of activity they were performing (e.g. answered emails; talked on telephone; read papers, entered data; performed analyses, attended meeting, etc) and whether that activity was computer-based or not. At the end of the questionnaire, they were also asked to indicate whether that day was a typical working day for them in order to make inferences regarding the entire working week.

To assess whether sitting vs standing patterns changed over time, the number of instances each week participants reported sitting rather than standing or being away from their desk, were calculated as a proportion of the total reported weekly occasions (total of 11 instances each week). These were averaged across participants and plotted to reveal potential trends over time. Similarly, to assess whether sitting vs standing patterns changed as a function of the time of day, the proportion of sitting instances occurring during each time (e.g. 9am; 10am etc) were calculated, averaged across study weeks and plotted. To assess whether sitting vs standing occasions differed as a function of the type of activity performed, chi-square analysis was performed on the percentage of computer based vs non-computer based activities reported as performed while sitting vs standing. Finally, the reasons participants reported for switching from sitting to standing and standing to sitting were classified according to their main theme and the frequency with which each main theme was reported was calculated.

Descriptive analysis of the weekly activity logs filled by participants allocated to the use of sit-stand desks revealed certain trends related to self-reported sitting and standing at work. Use of the desks did not appear to change over time: the average percentage of occasions during which participants reported sitting at their desks remained relatively stable over the study weeks (Figure 1). The time of day, however, seemed to play a role, with the average percentage of sitting occasions starting relatively high in the morning at the start of the working day, decreasing in the early afternoon around lunch and post-lunch time and steadily increasing in the late afternoon (Figure 2). In terms of the specific desk-related activities participants reported as performing (e.g. checking emails, data analysis, data entry, making calls etc), no clear pattern emerged. A significantly higher percentage, however, of non-computer-based activities, where reported during sitting occasions compared to standing occasions (sitting: 11% vs standing: 5.5%; χ^2^ (1) = 8.108, p<0.01).

The main reasons for switching between positions, can be seen in Tables 2 and 3.

**Table S2:** Reasons for switching from sitting to standing

| **Reason** | **Frequency** |
| --- | --- |
| Back ache and stiffness | 21% |
| Returning to desk after being away | 19.5% |
| Feeling guilty | 18% |
| Type of activity | 11% |
| Needing re-energizing | 8.5% |
| Conscious effort | 8.5% |
| Change of task | 6% |
| Social environment | 5% |
| Time of day | 2% |

**Table S3:** Reasons for Switching from standing to sitting

| **Reason** | **Frequency** |
| --- | --- |
| Tiredness | 57% |
| Leaving desk | 20% |
| Type of activity | 11% |
| Change of task | 8% |
| Social environment | 2% |

Although the above findings provide some insights into the circumstances under which the desks were used in standing and sitting mode, caution is warranted in drawing conclusions due to the limitations associated with the self-report measure used. Participants had to retrospectively report on their hourly activities and might have therefore not recalled them accurately. Furthermore, in analysing the data, unless specified otherwise by participants, it was assumed that activities reported for each completion day could be applied to the rest of the working week. The veracity of this assumption, however, is unknown.

**Table S4:** Acceptability of study procedures

| **Theme** | **Quotes** |
| --- | --- |
| **Acceptability of study** | *“it was overall a very positive experience*” (OW09)  “*Yeah, it was just an interesting kind of study*” (OW89) |
| **Acceptability of intervention** | *“I don’t want to give it (the desk) back”* (OW73)  *“I didn’t particularly want a standing desk, I'm quite happy sitting… I have stuck to using this standing desk and it’s been very good. Sometimes with these things you try something which is different and you didn’t necessarily want to try it but when you try it you experience it and you have a much better perspective*” (OW03) |
| **Acceptability of assessments** | “*It was all fine…the tests weren’t anything major…*” (OW15) |
| Acceptability of wearing monitors | “*The monitors are obviously going to be annoying, no matter what you do. But yeah, you can easily like shower and stuff in them so that’s all cool. One of the monitors did give me a bit of a rash this time but it didn’t last time so it might just be me. But yeah, otherwise, absolutely fine*” (OW15)  “*I got chaffing from the leg one…there was a degree of discomfort from that …*”(OW03)  “*you have to get used to them a little bit. If you wear these things on your chest it starts to get itchy a little bit after a while, it’s there only for a couple of days but it’s not. Its ok, it’s also not extremely comfortable…*” (OW09)  *So I had my follow-up assessment yesterday and I actually opted out of the monitors part of it, purely because they were just uncomfortable and quite a nuisance… Yeah, I mean mainly probably the chest one, we had quite a difficult time putting it on to start with, actually getting the positioning right, and it made me a bit more conscious about what I was wearing while I was wearing it, trying to cover it up*” (OW89) |
| Acceptability of food diary | *It’s difficult to keep up with your food diary”* (OW15)  “*The food diary was the hardest…it’s quite difficult to do because you want to try to be specific but then like for example we went to my mother-in-law’s house and she cooked food and I was like I’m not going to go and ask her exactly what she put in that, like I’m sorry, that’s not, for science but not that, so the food diary I thought was quite hard*” (OW47) |
| Acceptability of online work diary | *“But*  *“But yeah and filling in the survey, I guess, it’s not really a criticism, it’s fine because you’ve got to do your study and you need the data. I absolutely understand that but there is a lot of work associated with what we have to do”* (OW03).  *“…what I struggled with sometimes was with the weekly assessment…”* (OW73) |

1. [www.nhsemployers.org/~/media/Employers/Publications/Gender%20in%20the%20NHS.PDF](http://www.nhsemployers.org/~/media/Employers/Publications/Gender%20in%20the%20NHS.PDF) [↑](#footnote-ref-1)
2. [https://www.**rcp**london.ac.uk/file/2025/download?token=jNctVLKc](https://www.rcplondon.ac.uk/file/2025/download?token=jNctVLKc) [↑](#footnote-ref-2)
3. <http://content.digital.nhs.uk/catalogue/PUB22088/nhs-work-stat-july-2016-pdf.pdf> [↑](#footnote-ref-3)
4. [www.nhsemployers.org/~/media/Employers/Publications/Age%20in%20the%20NHS.PDF](http://www.nhsemployers.org/~/media/Employers/Publications/Age%20in%20the%20NHS.PDF). [↑](#footnote-ref-4)
5. http://content.digital.nhs.uk/catalogue/PUB21763/nhs-staff-earn-jun-16.pdf [↑](#footnote-ref-5)
6. http://www.nhs.uk/NHSEngland/thenhs/about/Pages/overview.aspx [↑](#footnote-ref-6)
7. Kazi A, Duncan M, Clemes S & Haslam C (2014). A survey of sitting time among UK employees. Occupational Medicine, 64 (7), 497-502. [↑](#footnote-ref-7)
8. Health Survey for England (2014). Adult obesity and overweight. http://content.digital.nhs.uk/catalogue/PUB19295/HSE2014-ch9-adult-obe.pdf [↑](#footnote-ref-8)
